# Expression of TIM-3 and Gal-9 Immune Checkpoints in Chronic Lymphocytic Leukemia: The Potential Role of Interleukin-27

**DOI:** 10.3390/cimb47110881

**Published:** 2025-10-23

**Authors:** Ewelina Wędrowska, Tomasz Wandtke, Bartosz Ulaszewski, Edyta Cichocka, Robert Dębski, Piotr Kopiński, Jan Styczyński, Grzegorz Przybylski

**Affiliations:** 1 Department of Lung Diseases, Neoplasms and Tuberculosis, Ludwik Rydygier Collegium Medicum in Bydgoszcz, Nicolaus Copernicus University in Toruń, 85-326 Bydgoszcz, Poland; tomasz.wandtke@cm.umk.pl (T.W.); mpkopins@cm.umk.pl (P.K.); gprzybylski@cm.umk.pl (G.P.); 2 Department of Genetics, Kazimierz Wielki University, 85-064 Bydgoszcz, Poland; ulaszewski@ukw.edu.pl; 3 Department of Hematology, L. Rydygier Provincial Polyclinical Hospital in Toruń, 87-100 Toruń, Poland; edytacichocka@gmail.com; 4 Department of Pediatric Hematology and Oncology, Ludwik Rydygier Collegium Medicum in Bydgoszcz, Nicolaus Copernicus University in Toruń, 85-094 Bydgoszcz, Poland; rdebski@cm.umk.pl (R.D.); jstyczynski@cm.umk.pl (J.S.)

**Keywords:** chronic lymphocytic leukemia, immune checkpoints, TIM-3, galectin-9, interleukin-27, T cell exhaustion

## Abstract

Background: Chronic lymphocytic leukemia (CLL) is characterized by malignant B lymphocyte accumulation and progressive immune dysfunction. The immune checkpoint molecule TIM-3 and its ligand galectin-9 (Gal-9) contribute to T cell exhaustion, impairing anti-tumour immunity. Interleukin-27 (IL-27) has pleiotropic immunomodulatory properties, but its impact on TIM-3 and Gal-9 expression in CLL remains unclear. Methods: Peripheral blood mononuclear cells (PBMCs) from 20 treatment-naive CLL patients were cultured with or without IL-27 (100 ng/mL) for 72 h. Flow cytometry assessed TIM-3 and Gal-9 expression on CD4^+^, CD8^+^, and CD19^+^ cells. Results: IL-27 stimulation significantly increased TIM-3 expression on CD8^+^ T cells (2.18 ± 0.32% vs. 3.09 ± 0.49%, *p* = 0.009), a hallmark of T cell exhaustion. IL-27 also modestly increased intracellular Gal-9 levels in total lymphocytes (93.91 ± 1.17% vs. 96.55 ± 0.67%, *p* = 0.005). Additionally, IL-27 reduced CD4^+^ T cell proportions (26.71 ± 4.19% vs. 22.01 ± 3.23%, *p* = 0.010). Although numerically modest, these changes may be biologically pertinent in the context of checkpoint-mediated CD8^+^ T-cell exhaustion. Conclusions: IL-27 may enhance immunosuppressive mechanisms in CLL by modulating immune checkpoint expression, potentially contributing to disease progression. These ex vivo findings in PBMCs from CLL patients indicate the IL-27-associated modulation of checkpoint expression under the conditions tested. In the absence of parallel healthy-donor controls, CLL specificity cannot be established in this study.

## 1. Introduction

Chronic lymphocytic leukemia (CLL) represents the most frequently diagnosed type of leukemia globally, constituting around 30% of all hematologic malignancies [1,2]. The incidence of CLL exhibits geographical variability, with the highest rates observed in Western countries, reaching approximately 5 cases per 100,000 individuals annually. In contrast, the disease is relatively rare in Asia. CLL is characterized by the accumulation of defective B lymphocytes in peripheral blood, bone marrow, and lymphoid tissues, leading to immune system dysfunction and clinical symptoms in more advanced stages of the disease. Genetic mutations, such as 13q deletion and overexpression of Bcl-2 family proteins, play a pivotal role in the pathogenesis of CLL by impairing apoptotic pathways in malignant cells [3,4].

In recent years, increasing attention has been devoted to studying the role of immune checkpoint mechanisms in the pathogenesis of CLL. Two key molecules in this context are the TIM-3 (T cell Immunoglobulin and Mucin domain-3) receptor and its ligand, galectin-9 (Gal-9). TIM-3 is expressed on the surface of activated CD4^+^ and CD8^+^ T lymphocytes, B lymphocytes, and other immune cells. Its interaction with Gal-9 reduces the effector activity of T lymphocytes by disrupting immunological synapse function. This process involves the phosphorylation of tyrosine residues in the cytoplasmic domain of TIM-3, leading to the release of the BAT-3 (HLA-B associated transcript-3 isoform b) protein and activation of signalling cascades that increase intracellular calcium ion levels. This results in the cellular anergy and apoptosis of T lymphocytes [5,6,7,8,9]. This process exacerbates T cell exhaustion, a state characteristic of the tumour microenvironment in CLL, which plays a crucial role in suppressing anti-tumour responses [10].

Interleukin-27 (IL-27), a member of the IL-12 cytokine family, is a heterodimeric molecule composed of p28 and EBI3 (Epstein–Barr virus induced gene 3) subunits. IL-27 exhibits multifaceted and context-dependent effects. Initially recognized as a pro-inflammatory cytokine, IL-27 promotes Th1-type immune responses while concurrently suppressing Th2, Th17, and Treg activity. However, IL-27 has also been shown to exhibit immunosuppressive properties, including the ability to induce IL-10 production [11,12,13]. Interleukin-27 (IL-27) is a cytokine that can exhibit both anti-tumour and pro-tumour activities depending on the cancer type. Elevated levels of IL-27 have been reported in certain malignancies, including melanoma and acute myeloid leukemia (AML) [14,15,16]. Based on these observations, it appears that IL-27 may exert different effects depending on the tumour type; thus, there is a need to thoroughly investigate its role in chronic lymphocytic leukemia (CLL). To date, information on IL-27’s impact in CLL remains limited. Several studies have indicated reduced IL-27 levels in CLL patients, which may suggest an antitumor role of this cytokine. For instance, Pagano et al. reported a significant and consistent decrease in serum IL-27 levels as the disease progresses, supporting its potential anti-tumour effect in CLL [17]. Similarly, Manouchehri-Doulabi et al. observed lower IL-27 and receptor expression in peripheral blood mononuclear cells of CLL patients compared to healthy controls, alongside enhanced natural killer (NK) cell activity and decreased B cell proliferation [4]. In this study, we aimed to investigate whether IL-27 stimulation affects the expression of immune checkpoint molecules TIM-3 and Gal-9, thereby elucidating its potential anti-tumour role in CLL. Previous studies suggest that IL-27 plays a multifaceted role in tumourigenesis. In some cases, it has demonstrated anti-tumour effects, such as limiting the activity of M2 macrophages and stimulating effector NK and Tc lymphocytes. Simultaneously, unfavourable effects have been observed, including increased expression of immunosuppressive molecules like PD-L1 (Programmed Death-Ligand 1) and IDO (Indoleamine 2,3-dioxygenase), which impair immune responses in the tumour microenvironment [18,19,20].

Currently, data on the impact of IL-27 on the expression of TIM-3 and Gal-9 in the context of CLL are lacking. This study addresses this knowledge gap by examining how IL-27 stimulation modulates immune checkpoint expression on lymphocyte subsets in CLL patients, potentially revealing new therapeutic mechanisms.

## 2. Materials and Methods

### 2.1. Patients

This study included 20 patients from the Haematology Department of the Provincial Polyclinical Hospital in Toruń named after L. Rydygier, Poland. All participants were diagnosed with CLL between January and December 2023, according to the current diagnostic criteria established by the Polish Society of Haematologists and Transfusionists [21]. The diagnosis was comprehensively confirmed through clinical evaluation, detailed blood counts, morphological assessments, and immunophenotyping. Notably, none of the patients had received any prior treatment for CLL.

To ensure a well-defined and homogeneous study cohort, strict exclusion criteria were implemented. Patients were excluded if they presented with any of the following: a current or prior diagnosis of other neoplastic disorders; acute or chronic inflammatory conditions, particularly those of autoimmune origin; diabetes mellitus; pregnancy; abnormal results from liver, pancreatic, renal, or cardiac function tests, whether current or historical; use of immunosuppressive drugs within the six months prior to enrolment.

The study protocol received formal approval from the Local Bioethics Committee of Nicolaus Copernicus University in Toruń, Ludwik Rydygier Collegium Medicum in Bydgoszcz. Written, informed consent was obtained from all participants prior to inclusion, ensuring their voluntary and fully informed participation in the study.

The rigorous patient selection process and adherence to ethical guidelines were designed to ensure the reliability and scientific validity of the study outcomes, reinforcing the robustness of its methodology.

### 2.2. Blood Samples

A 10 mL sample of peripheral blood was obtained from each participant via venipuncture of the antecubital vein. The blood was collected into BD Vacutainer tubes containing lithium heparin as an anticoagulant. Immediately after collection, the samples were transported to the laboratory under controlled conditions, maintaining a temperature range of 2–8 °C to ensure the preservation of cellular and molecular integrity [22].

### 2.3. PBMCs Isolation

Heparinized blood was aliquoted into 15 mL tubes and diluted 1:1 with calcium- and magnesium-free phosphate-buffered saline (PBS) (Corning, cat. no. 21-040-CV, Corning, NY, USA). The diluted sample was carefully layered onto a Histopaque^®^-1077 density gradient (Sigma-Aldrich, cat. no. 10771-6 × 100ML, Darmstadt, Germany) at a 4:3 ratio. Following this, the samples were centrifuged at 450× *g* for 25 min at room temperature using Sigma Laborzentrifugen GmbH centrifuge (Osterode, Germany). The interphase, containing peripheral blood mononuclear cells (PBMCs), was meticulously collected and transferred into fresh tubes. The PBMCs were then washed twice with PBS, each wash followed by centrifugation at 300× *g* for 6 min at room temperature (RT) [23].

The purified PBMCs were subsequently utilized for in vitro cell culture experiments.

### 2.4. Cell Cultures

To investigate the effects of IL-27 on the subpopulations of CD4^+^ and CD8^+^ lymphocytes as well as B lymphocytes, PBMCs isolated from CLL patients were cultured in the presence or absence of IL-27.

Cell cultures were maintained in RPMI-1640 medium supplemented with L-glutamine (Corning, cat. no. 10-040-CV, Corning, NY, USA) and 10% heat-inactivated, sterile-filtered fetal bovine serum (Sigma-Aldrich, cat. no. F9665-500ML, Darmstadt, Germany). The medium was further supplemented with 100 U/mL penicillin, 100 µg/mL streptomycin, and 0.25 µg/mL amphotericin B (Antibiotic-Antimycotic, Gibco, cat. no. 15240062, Grand Island, NE, USA). For conditions involving IL-27, recombinant human IL-27 (PeproTech, cat. no. 200-38-100UG, Cranbury, NJ, USA) was added at a final concentration of 100 ng/mL [24].

Cultures were seeded at a density of 1 × 10^5^ cells/mL in culture flasks and incubated for 72 h at 37 °C with 5% CO_2_ and 95% humidity [24].

### 2.5. Flow Cytometry Staining and Analysis

Following completion of the cell cultures, suspensions were centrifuged (300× *g*, 6 min, RT) and resuspended in PBS. Surface staining was performed with fluorochrome-conjugated monoclonal antibodies for 30 min at room temperature (RT) protected from light, followed by a wash in PBS + 0.1% NaN_3_ (300 g, 8 min, RT) and fixation in 2% paraformaldehyde. The surface panel included: CD45-PerCP-Cy5.5 (clone HI30; BD Biosciences, cat. 564105, Franklin Lakes, NJ, USA), CD3-PE (HIT3α; BD, 555340, Franklin Lakes, NJ, USA), CD4-FITC (RPA-T4; BD, 561842, Franklin Lakes, NJ, USA), CD8-APC (RPA-T8; BD, 561953, Franklin Lakes, NJ, USA), CD19-PE-Cy7 (SJ25C1; BD, 557835, Franklin Lakes, NJ, USA), and TIM-3-PE (7D3; BD, 563422, Franklin Lakes, NJ, USA). Appropriate isotype controls for each monoclonal antibody were also used where available.

For intracellular Gal-9, after surface staining and fixation, cells (1.5 × 10^5^ per test) were permeabilized with BD FACS Permeabilizing Solution (1×; BD Biosciences, cat. 340973, Franklin Lakes, NJ, USA) for 10 min at RT in the dark, washed in PBS + 0.1% NaN_3_ (500 g, 5 min), and stained with anti-Gal-9 (clone 9M1-3, PerCP-Cy5.5; BioLegend, cat. 348910, San Diego, CA, USA) prepared in permeabilization buffer for 30 min at RT protected from light. Samples were then washed as described above and resuspended in PBS for acquisition. Surface markers were stained prior to fixation to preserve epitope integrity. Flow cytometry staining was performed strictly according to the manufacturers’ recommendations for all conjugated antibodies and reagents to ensure optimal specificity and sensitivity.

Data were acquired on a BD FACSCanto II and analyzed in Kaluza (Beckman Coulter, Brea, CA, USA). The gating hierarchy was CD45^+^ leukocytes → lymphocytes (FSC/SSC) → singlets (FSC-A vs. FSC-H) → CD3 → CD4/CD8 and CD19, with TIM-3 and Gal-9 quantified within each subset. At least 10,000 lymphocyte events per sample were evaluated. Results are reported as percentages of the parental gate, with median fluorescence intensity (MFI) provided where indicated.

Compensation was computed from single-stained controls acquired under identical settings when available; otherwise, instrument-standard compensation for the configured panel was applied and verified against channel spillover patterns in the acquired data. Positivity thresholds were anchored to internal negative populations within the relevant parent gates and to unstained controls; where isotype controls were available, they informed non-specific binding. In the absence of full FMO panels, gates for low-intensity targets (e.g., Gal-9) were set conservatively and conclusions were tempered accordingly.

To minimize potential bias introduced by non-viable cells, all samples were gated based on FSC/SSC parameters to exclude debris and cells with abnormal morphology. In addition, cell viability was routinely assessed in parallel samples using trypan blue exclusion test. Viability consistently exceeded 97% at day 3 of culture, indicating that the analyzed populations largely represented live cells. Flow cytometry staining and gating strategies were performed according to best practice guidelines [25] and previously established protocols for immune checkpoint detection on human PBMCs [26].

### 2.6. Statistical Analysis

Fluorescence data underwent standard transformations (biexponential or arcsinh, as appropriate) before statistical analysis. Statistical analyses were performed using Statistica 13 (StatSoft, Krakow, Poland). Data normality was assessed using the Shapiro–Wilk test. Comparisons between IL-27− treated and untreated conditions were made using the Wilcoxon signed-rank test for paired samples. Where applicable, effect sizes with 95% confidence intervals were reported alongside *p*-values to facilitate assessment of biological relevance. Statistical significance was defined as *p* < 0.05.

## 3. Results

### 3.1. Experimental Design and Approach

To investigate IL-27 effects on immune checkpoint expression in CLL, we employed an in vitro model using PBMCs from 20 treatment-naive CLL patients. Cells were cultured for 72 h with or without IL-27 (100 ng/mL), followed by comprehensive flow cytometric analysis of lymphocyte populations and immune checkpoint molecule expression.

### 3.2. IL-27 Effects on Lymphocyte Population Distribution

IL-27 treatment significantly altered lymphocyte subset proportions in CLL patients (Table 1). The most pronounced effect was observed in CD4^+^ T helper (Th) cells, which decreased from 26.71 ± 4.19% (median: 23.59%) to 22.01 ± 3.23% (median: 21.77%) following IL-27 exposure (*p* = 0.010). Although numerically modest (≈ −4.7 percentage points on average), this CD4^+^ reduction may be biologically pertinent to T-cell balance in CLL; interpretation remains cautious given study limitations.

Additionally, total CD3^+^ T lymphocyte proportions significantly declined from 43.26 ± 5.94% (median: 42.57%) to 36.85 ± 5.48% (median: 40.18%) (*p* = 0.039) in the presence of IL-27. No statistically significant changes were observed in CD8^+^ T cytotoxic (Tc) lymphocytes or CD19^+^ B lymphocytes (*p* > 0.05).

**Figure 1:**
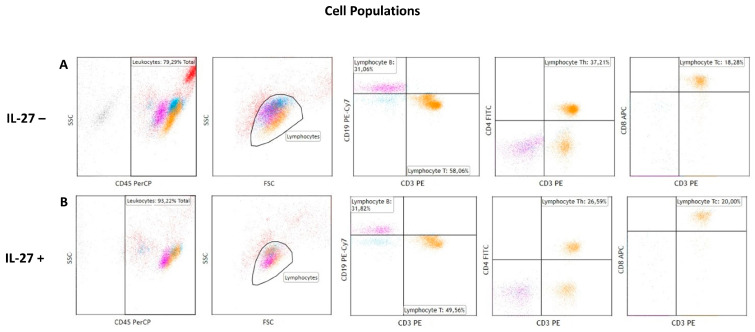
The gating strategy involved the identification of the leukocyte population based on CD45 expression and side scatter (SSC). Lymphocytes were subsequently gated using forward and side scatter properties. B cells (magenta) were identified by CD19^+^/CD3^−^ staining. CD4^+^ (Th) and CD8^+^ (Tc) T cell subsets (orange) were distinguished using CD3^+^/CD4^+^ and CD3^+^/CD8^+^ staining, respectively. Results are expressed as percentages of the total lymphocyte population. A minimum of 10,000 events was analyzed per sample. The red and blue colors represent the remaining CD45^+^ cell populations, e.g. monocytes. (**A**) Gating results of cellular populations in the experiment without IL-27 exposure. (**B**) Gating results of cellular populations in the experiment with IL-27 exposure.

**Figure 2:**
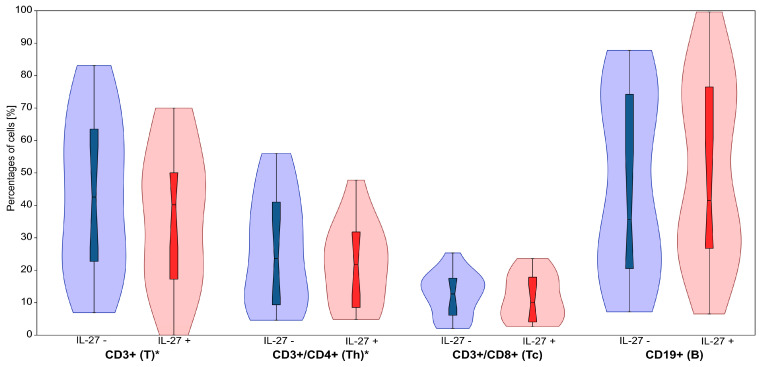
The effect of IL-27 on the percentages of T (CD3^+^), helper T (CD3^+^/CD4^+^, Th), cytotoxic T (CD3^+^/CD8^+^, Tc), and B (CD19^+^) lymphocytes. Violin plots display the distribution of cell percentages in IL-27− untreated (IL-27−) and IL-27− treated (IL-27+) conditions. Blue represents IL-27− untreated samples, while red represents IL-27− treated samples. The central boxplots within each violin indicate the median and interquartile range. * for *p* < 0.05.

These findings suggest that IL-27 predominantly affects the CD4^+^ T cell compartment, pointing toward its complex role in shaping immune balance in CLL. However, whether these effects reflect a pro-inflammatory or immunosuppressive outcome requires further investigation. A representative gating strategy is shown in Figure 1. Further details on the observed differences are provided in Table 1 and Figure 2.

### 3.3. IL-27 Upregulates TIM-3 on CD8^+^ T Cells

**Figure 3:**
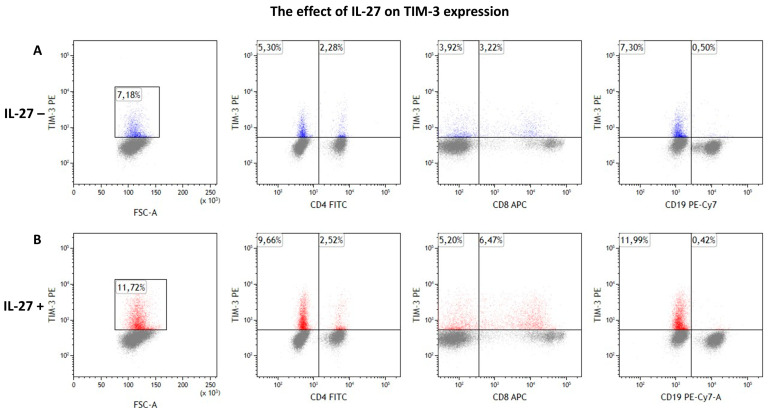
(**A**) TIM-3 expression in the experiment without IL-27 exposure. From left to right: total TIM-3 expression in the lymphocyte population, followed by TIM-3 expression in CD4^+^, CD8^+^, and CD19^+^ cell subsets. (**B**) TIM-3 expression in the experiment with IL-27 exposure. From left to right: total TIM-3 expression in the lymphocyte population, followed by TIM-3 expression in CD4^+^, CD8^+^, and CD19^+^ cell subsets. TIM-3^+^ lymphocytes are shown in blue in unstimulated IL-27 cultures and in red in stimulated cultures, whereas grey represents lymphocytes lacking TIM-3 expression in both conditions. The lymphocyte population was defined based on the gating strategy shown in Figure 1. Results are expressed as percentages of the total lymphocyte population. A minimum of 10,000 events was analyzed per sample.

**Figure 4:**
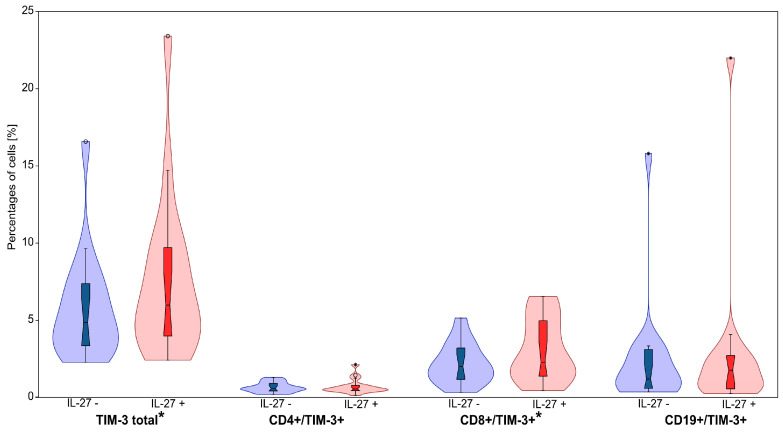
The effect of IL-27 on the percentages of TIM-3-expressing lymphocyte cells, including total TIM-3^+^ cells, CD4^+^/TIM-3^+^, CD8^+^/TIM-3^+^ and CD19^+^/TIM-3^+^ cells. Violin plots display the distribution of cell percentages in IL-27− untreated (IL-27−) and IL-27− treated (IL-27+) conditions. Blue represents IL-27− untreated samples, while red represents IL-27− treated samples. The central boxplots within each violin indicate the median and interquartile range. * for *p* < 0.05.

Flow cytometric analysis revealed significant IL-27-induced changes in TIM-3 expression patterns (Table 2, Figure 3 and Figure 4). Most notably, IL-27 exposure significantly increased TIM-3 expression on CD8^+^ T cells in CLL patients. The CD8^+^/TIM-3^+^ population expanded from 2.18 ± 0.32% (median: 2.02%) to 3.09 ± 0.49% (median: 2.27%) following IL-27 treatment (*p* = 0.009). Although numerically modest (~+0.91 percentage points), this increase may be biologically pertinent in the context of checkpoint-mediated CD8^+^ T-cell exhaustion.

Correspondingly, total lymphocyte TIM-3 expression increased from 5.77 ± 0.85% (median: 4.86%) to 7.54 ± 1.27% (median: 5.99%) after IL-27 exposure (*p* = 0.011), likely reflecting the contribution of CD8^+^ T cells. No statistically significant differences in TIM-3 expression were observed in CD4^+^ T cells or CD19^+^ B cells (*p* > 0.05), indicating specificity of the IL-27 effect for the CD8^+^ T cell compartment.

Although the magnitude of change was modest, these findings suggest that IL-27 may contribute to immunosuppressive mechanisms in CLL by selectively enhancing TIM-3 expression on CD8^+^ T cells.

### 3.4. IL-27 Modestly Increases Intracellular Gal-9 Expression

**Figure 5:**
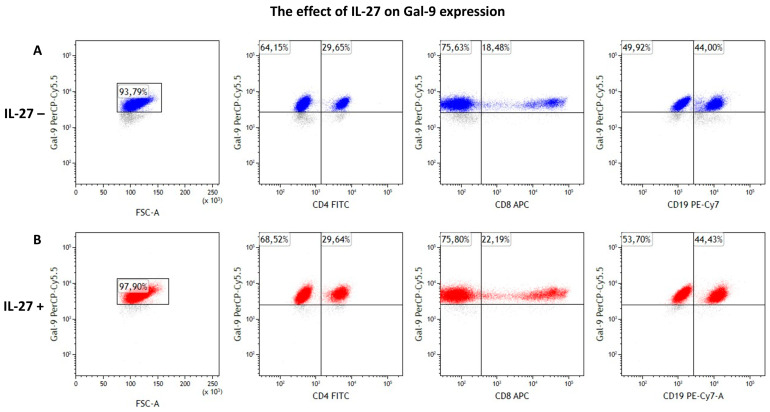
(**A**) Gal-9 expression in the experiment without IL-27 exposure. From left to right: total Gal-9 expression in the lymphocyte population, followed by Gal-9 expression in CD4^+^, CD8^+^, and CD19^+^ cell subsets. (**B**) Gal-9 expression in the experiment with IL-27 exposure. From left to right: total Gal-9 expression in the lymphocyte population, followed by Gal-9 expression in CD4^+^, CD8^+^, and CD19^+^ cell subsets. Gal-9^+^ lymphocytes are shown in blue in unstimulated IL-27 cultures and in red in stimulated cultures, whereas grey represents lymphocytes lacking Gal-9 expression in both conditions The lymphocyte population was defined based on the gating strategy shown in Figure 1. Results are expressed as percentages of the total lymphocyte population. A minimum of 10,000 events was analyzed per sample.

**Figure 6:**
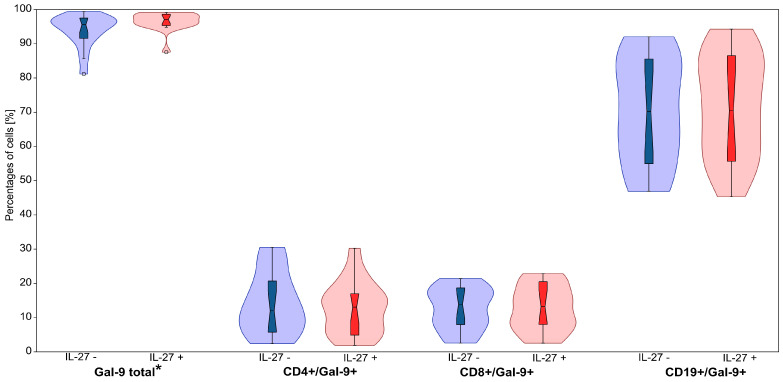
The effect of IL-27 on the percentages of Gal-9− expressing lymphocyte cells, including total Gal-9^+^ cells, CD4^+^/Gal-9^+^, CD8^+^/Gal-9^+^ and CD19^+^/Gal-9^+^. Violin plots display the distribution of cell percentages in IL-27− untreated (IL-27−) and IL-27− treated (IL-27+) conditions. Blue represents IL-27− untreated samples, while red represents IL-27− treated samples. The central boxplots within each violin indicate the median and interquartile range. * for *p* < 0.05.

IL-27 treatment significantly increased intracellular Gal-9 expression in CLL patients (Table 3, Figure 5 and Figure 6). The percentage of Gal-9^+^ lymphocytes rose from 93.91 ± 1.17% (median: 95.58%) to 96.55 ± 0.67% (median: 97.06%) (*p* = 0.005). Although modest (≈+2.6 percentage points on average), this shift may be biologically pertinent in a system with high baseline Gal-9; conclusions remain conservative.

No statistically significant changes were detected within CD4^+^, CD8^+^, or CD19^+^ subsets (*p* > 0.05).

Given the high baseline Gal-9 expression, the observed increase at the total lymphocyte level was modest. A representative flow cytometry gating strategy and expression profiles are shown in Figure 5 and Figure 6.

## 4. Discussion

Chronic lymphocytic leukemia remains a therapeutic challenge, with immune dysfunction mechanisms playing critical roles in disease progression [27,28,29,30,31]. Immune checkpoint molecules like TIM-3 and Gal-9 regulate interactions between malignant cells and the tumour microenvironment, representing potential therapeutic targets [8,9,10,32]. The role of IL-27 in CLL has remained largely unexplored, particularly regarding its effects on checkpoint expression—a significant knowledge gap—given IL-27’s dual nature as both pro-inflammatory and immunosuppressive cytokine [11,12,33,34].

This study provides first evidence that IL-27 modulates immune checkpoint expression in CLL patients, specifically demonstrating increased TIM-3 expression on CD8^+^ T cells and modest upregulation of intracellular Gal-9. While the observed changes were modest in magnitude, they were consistent across all patients and statistically significant, suggesting biological relevance in CLL pathophysiology.

Our study revealed that IL-27 stimulation in CLL patients results in a significant overall increase in TIM-3 expression within the lymphocyte population, primarily driven by statistically significant upregulation on CD8^+^ T cells. The elevated TIM-3 expression on CD8^+^ T cells is widely recognized as a hallmark of cellular exhaustion, a deleterious phenotype associated with increased susceptibility to apoptosis and diminished antitumor immune responses [35,36,37,38,39]. These findings highlight a potentially unfavourable role for IL-27 in the immunopathology of CLL. TIM-3, originally identified as a marker of terminally differentiated Th1 cells, functions as a crucial negative regulator that limits excessive immune activation under physiological conditions. However, in the tumour context, persistent TIM-3 expression creates a state of functional unresponsiveness that favours tumour escape from immune surveillance [8,32,35].

The role of IL-27 in CLL remains a subject of debate, as the literature presents conflicting evidence. Pagano et al. demonstrated in a murine model that IL-27 deficiency promoted a more immunosuppressive tumour microenvironment and accelerated CLL progression; however, their study did not specifically address the impact of IL-27 on TIM-3 expression [17]. In contrast, supporting our findings, several reports have described immunosuppressive effects of IL-27, including its role in driving TIM-3 upregulation [18,40,41].

Our findings align with mechanistic studies demonstrating IL-27’s ability to induce TIM-3 expression through NFIL3 and T-bet transcription factors [33,40,42] both of which are regulated by STAT1/3 signalling [40]. IL-27 promotes NFIL3 expression, which directly upregulates TIM-3, while cooperative NFIL3 and T-bet action facilitates chromatin remodelling at the TIM-3 promoter [40]. Given T-bet’s important role in CD8^+^ T cell differentiation and its IL-27-dependent expression [43,44,45,46], similar mechanisms likely underlie our observations in CLL patients.

Supporting our results, DeLong et al. demonstrated IL-27-induced TIM-3 upregulation on CD8^+^ T cells in murine models [47], while Chiba et al. reported increased TIM-3 expression on myeloid cells following IL-27 stimulation [48]. To our knowledge, this is the first study demonstrating IL-27-induced TIM-3 upregulation specifically in CLL patients. Even small increments in TIM-3 on CD8+ cells can contribute toward exhaustion thresholds over time; the present dataset supports association rather than causality.

Our observations are consistent with previous reports of elevated Gal-9 expression in CLL B lymphocytes, which aligns with findings from Wdowiak et al., who reported increased levels of soluble Gal-9 in the serum of CLL patients [49]. These data indirectly support our results, suggesting that leukemic B lymphocytes may represent a cellular source of Gal-9 in CLL. Furthermore, it is plausible that these cells have the capability to release Gal-9 into the extracellular environment. This phenomenon is of particular concern, as elevated levels of Gal-9 in CLL patients have been positively correlated with advanced disease stages and are associated with poor prognostic outcomes [49,50]. At the total lymphocyte level, the IL-27-induced Gal-9 shift was modest against a high baseline and should be interpreted cautiously.

Shedding of Gal-9 from the surfaces of leukemic cells has been well-documented in studies on AML, where extracellular Gal-9 has been shown to activate TIM-3 signalling pathways, including in NK cells [51,52]. It is reasonable to hypothesize that a similar mechanism operates in CLL, with leukemic B lymphocytes as the likely source. Notably, Gal-9 exists in multiple isoforms with distinct binding affinities for TIM-3, and its extracellular release can create localized immunosuppressive niches that extend beyond direct cell–cell interactions, potentially affecting distant immune effector cells within the tumour microenvironment [53,54,55,56]. In our study, stimulation with IL-27 induced a modest yet statistically significant increase in intracellular Gal-9 expression across the lymphocyte population in CLL patients. These findings, in light of previously reported data, provide further evidence for the potentially deleterious role of IL-27 in the context of CLL [11,18,57].

In CLL, Gal-9 binding to its receptor TIM-3 on Th1 and Treg cells has been shown to suppress IL-2 production, inhibit Th1 cell expansion, and induce Th1 apoptosis [53,57,58]. Additionally, Gal-9 interaction with TIM-3 on Treg cells promotes the proliferation of these cells, enhances the secretion of the immunosuppressive cytokine IL-10, and suppresses the production of pro-inflammatory cytokines such as IFN-γ and TNF-α (Tumor Necrosis Factor α) by Th1 cells. These immunosuppressive effects have also been observed in CLL patients [57,58,59].

These findings provide novel insights into the role of Gal-9 and its regulation by IL-27 in CLL, suggesting a mechanistic link between increased Gal-9 expression, immunosuppressive microenvironment modulation, and disease progression.

In light of the observations made, IL-27 appears to induce a phenotype characteristic of exhaustion in CD8^+^ cells in patients with CLL, as evidenced by the elevated expression of TIM-3 on their surface. This phenomenon is closely linked to an increased likelihood of apoptosis within this cell population. It is important to note that TIM-3 expression is not restricted to cytotoxic T lymphocytes but is also observed on NK cells. Previous studies have reported that NK cells in patients with CLL exhibit higher baseline TIM-3 expression compared to healthy individuals [6,60]. Therefore, the observed increase in TIM-3 expression in response to IL-27 in this study may affect CD8^+^ T cells, NK cells, or both populations. Further in-depth investigations are necessary to determine the specific cellular targets of IL-27 within the CD8^+^ compartment.

Regardless, the observed effect of IL-27 has an unfavourable impact on the anti-tumour response, particularly in CLL, where both CD8^+^ T cells and NK cells play a critical role in eliminating malignant cells. Moreover, the modest but statistically significant increase in Gal-9 expression in lymphocytes induced by IL-27 may further contribute to its deleterious effects in CLL. This is especially pertinent given that Gal-9 expression in CLL predominantly localizes to malignant B lymphocytes, which constitute the tumour cells [49,53].

Taken together, statistically significant but modest shifts can still be biologically meaningful in CLL, yet conclusions are deliberately tempered by methodological constraints outlined in this study.

Additionally, IL-27 may exert broader immunosuppressive effects on the anti-tumour response than those described here. For instance, TIM-3 expression has been detected on dendritic cells [61,62], raising the possibility that IL-27 might influence other cell populations and modulate the expression of immune checkpoint molecules on their surface. Such effects could collectively impair the efficiency of anti-tumour immunity in CLL patients.

Furthermore, there is evidence from the literature indicating that AML cell lines exposed to IL-27 exhibit increased proliferation rates and acquire chemoresistance [58,63]. Similarly, IL-27 has been consistently reported to exert immunosuppressive effects by stimulating IL-10 expression and inducing other immune checkpoints beyond TIM-3, even in non-lymphocyte cell populations [11,12,13,19,20,40,64].

### 4.1. Study Limitations and Future Directions

Several important limitations should be acknowledged. This study lacks a parallel healthy donor control arm; therefore, CLL specificity versus general IL-27 effects on PBMCs cannot be determined under the present design. Nevertheless, our design was guided by earlier studies reporting decreased IL-27 levels in CLL patients, which had been interpreted as suggestive of an anti-tumour role for this cytokinę [4,17]. We therefore aimed to examine the direct impact of IL-27 within CLL-derived PBMCs, focusing on paired comparisons of unstimulated and IL-27–stimulated cells from the same patients. This intra-cohort approach enabled us to capture cytokine-dependent changes independently of baseline differences between patients and healthy controls. Future work including both CLL and healthy donor samples will be essential to confirm and extend these observations

The in vitro culture system based on PBMCs, cannot fully reproduce the complexity of the CLL microenvironment, including stromal support, cytokine networks, and hypoxic niches. Consequently, the generalizability of our findings to the in vivo context may be limited. Nevertheless, this reductionist approach enabled us to delineate the direct effects of IL-27 on CLL-derived cells, independent of additional confounding factors. The microenvironment may significantly influence or modulate these effects, and future studies using co-culture systems or in vivo models will be required to validate and extend our findings. While simplified, our in vitro system offers the advantage of isolating the direct effects of IL-27 on CLL cells, allowing a clearer assessment of cytokine-dependent modulation of TIM-3 and Gal-9 without interference from complex microenvironmental factors. Marked reductions in FSC/SSC events were observed under IL-27 treatment in some samples, but a viability dye was not used; thus, proportional shifts may partly reflect differential survival rather than purely phenotypic changes. Future studies should incorporate live/dead staining and consider the inclusion of stromal support to better distinguish direct IL-27 effects on cell survival from those on phenotype.

While the 72 h incubation timepoint represents a standard approach for cytokine studies [34,45] early kinetics (2–48 h) were not assessed. Future investigations should employ a time-course design to understand the temporal dynamics of IL-27 effects on checkpoint expression.

One limitation of our study is that we did not perform ELISA measurements of IL-27 serum levels in our patient cohort due to sample availability constraints. Future studies including such measurements could provide further insight into the role of IL-27 in CLL pathogenesis and its potential as a therapeutic target.

Flow cytometry controls were incomplete for all markers/timepoints (no universal FMO set and no fixable viability dye), which limits the precision of positivity thresholds for dim markers and the attribution of proportional shifts to survival versus phenotype; we therefore used conservative gates for low-intensity signals and tempered claims accordingly in the Results and Discussion.

Orthogonal validation by qPCR or Western blot was not performed within this protocol; therefore, claims have been moderated to avoid overinterpretation of flow cytometry data alone.

Future studies should investigate additional TIM-3 ligands beyond Gal-9, examine transcription factors like NFIL3 and T-bet, and include comprehensive apoptotic markers to better understand IL-27’s mechanistic effects. The clinical relevance of these in vitro findings requires validation in larger patient cohorts with healthy controls and correlation with disease parameters.

### 4.2. Clinical Implications

The finding that IL-27 enhances immunosuppressive checkpoint expression suggests caution regarding IL-27-based therapies in CLL. However, these results also highlight potential therapeutic opportunities. Combination approaches using IL-27 alongside TIM-3 or PD-1 checkpoint inhibitors might achieve superior outcomes compared to monotherapy approaches, which have shown limited efficacy in CLL [65,66].

From a clinical perspective, the IL-27-induced increase in TIM-3 and Gal-9 expression observed in this study may have important implications for disease progression and prognosis in CLL. Previous studies have demonstrated that elevated TIM-3 expression on CD8+ T cells and NK cells correlates with more advanced Rai and Binet stages, reduced cytotoxic activity, and shorter time to first treatment [67]. Similarly, increased Gal-9 expression has been associated with unfavourable prognostic markers, including high-risk cytogenetic aberrations such as del (17p) and TP53 mutations, as well as elevated β2-microglobulin levels [68]. Given that both TIM-3 and Gal-9 contribute to T cell exhaustion and immunosuppressive signalling, our findings suggest that IL-27 may enhance these pathways and thereby promote immune dysfunction related to advanced disease. Although clinical outcomes were not directly assessed in our cohort, these molecular patterns are consistent with phenotypes observed in CLL patients with poor response to therapy, particularly to chemoimmunotherapy or immune checkpoint blockade. Future longitudinal studies integrating clinical and molecular data will be essential to validate these associations and determine whether IL-27-driven TIM-3/Gal-9 modulation can serve as a biomarker of disease aggressiveness or treatment resistance.

## 5. Conclusions

This study provides the first direct evidence that interleukin-27 (IL-27) modulates immune checkpoint pathways in chronic lymphocytic leukemia (CLL). Using ex vivo PBMC cultures from treatment-naive CLL patients, we demonstrated that IL-27 stimulation induces a statistically significant increase in TIM-3 expression on CD8+ T lymphocytes (from 2.18 ± 0.32% to 3.09 ± 0.49%, *p* = 0.009) and elevates total TIM-3+ lymphocyte frequencies (from 5.77 ± 0.85% to 7.54 ± 1.27%, *p* = 0.011). Although modest in magnitude, these shifts are biologically meaningful, as TIM-3 upregulation on CD8+ T cells is a hallmark of T cell exhaustion, a state associated with reduced cytotoxic function and impaired tumour surveillance.

We also observed a statistically significant increase in intracellular Gal-9 expression in total lymphocytes (from 93.91 ± 1.17% to 96.55 ± 0.67%, *p* = 0.005), indicating that IL-27 may amplify immunosuppressive signalling through both receptor and ligand components of the TIM-3/Gal-9 axis. Additionally, IL-27 exposure reduced the proportion of CD4+ T cells, further suggesting a shift in T cell compartment balance toward an immunosuppressive phenotype.

Taken together, these findings support a model in which IL-27 contributes to the establishment or reinforcement of an immunosuppressive microenvironment in CLL through the upregulation of TIM-3 on effector T cells and enhancement of Gal-9 expression. These mechanisms may facilitate immune evasion by leukemic cells and promote disease progression.

From a translational perspective, our results highlight the IL-27/TIM-3/Gal-9 axis as a potential therapeutic target. Interventions combining IL-27 modulation with immune checkpoint inhibition, particularly TIM-3 blockade, may enhance the effectiveness of immunotherapy in CLL. Furthermore, TIM-3 and Gal-9 levels could serve as candidate biomarkers for monitoring disease activity and immune dysfunction.

Future studies should validate these observations in larger patient cohorts with matched healthy controls, integrate longitudinal clinical outcomes, and dissect the underlying molecular mechanisms, including the role of NFIL3 and T-bet signalling. Such efforts may provide new insights into the contribution of IL-27 to CLL pathophysiology and inform the development of targeted immunotherapeutic strategies.

## Figures and Tables

**Table 1:** Comparison of the percentages of T cells (including Th and Tc subpopulations) and B cells in CLL patients between cell cultures with and without IL-27 exposure.

Cell Population	Cell Culture	Mean	SD	SEM	Median	Min.	Max.	Test Statistic	*p*-Value
CD3^+^ (T)	IL-27–	43.26	24.47	5.94	42.57	6.92	83.08	2.068 ^W^	0.039
	IL-27+	36.85	21.90	5.48	40.18	0.01	69.99		
CD3^+^/CD4^+^ (Th)	IL-27–	26.71	17.26	4.19	23.59	4.60	56.01	2.585 ^W^	0.010
	IL-27+	22.01	12.94	3.23	21.77	4.85	47.80		
CD3^+^/CD8^+^ (Tc)	IL-27–	12.69	6.74	1.63	12.70	2.09	25.33	0.931 ^W^	0.352
	IL-27+	11.64	7.19	1.80	10.10	2.65	23.59		
CD19^+^ (B)	IL-27–	46.82	28.57	6.93	35.65	7.18	87.80	0.686 ^W^	0.492
	IL-27+	51.02	28.40	6.89	41.55	6.54	99.60		

SD—Standard Deviation, SEM—Standard Error of Mean, ^W^—Wilcoxon test.

**Table 2:** Comparison of the percentages of TIM-3^+^, CD4^+^/TIM-3^+^, CD8^+^/TIM-3^+^ and CD19^+^/TIM-3^+^ cells in CLL patients between cell cultures with and without IL-27 exposure.

Cell Population	Cell Culture	Mean	SD	SEM	Median	Min.	Max.	Test Statistic	*p*-Value
TIM-3^+^ total	IL-27–	5.77	3.50	0.85	4.86	2.28	16.58	2.533 ^W^	0.011
	IL-27+	7.54	5.24	1.27	5.99	2.42	23.41		
CD4^+^/TIM-3^+^	IL-27–	0.66	0.33	0.08	0.58	0.19	2.28	0.781 ^W^	0.435
	IL-27+	0.71	0.48	0.12	0.55	0.13	2.52		
CD8^+^/TIM-3^+^	IL-27–	2.18	1.32	0.32	2.02	0.32	5.15	2.627 ^W^	0.009
	IL-27+	3.09	2.04	0.49	2.27	0.44	6.55		
CD19^+^/TIM3^+^	IL-27–	2.41	3.62	0.88	1.20	0.36	15.80	0.213 ^W^	0.831
	IL-27+	2.82	5.06	1.23	1.77	0.25	21.99		

SD—Standard Deviation, SEM—Standard Error of Mean, ^W^—Wilcoxon test.

**Table 3:** Comparison of the percentages of Gal-9^+^, CD4^+^/Gal-9^+^, CD8^+^/Gal-9^+^ and CD19^+^/Gal-9^+^ cells in CLL patients between cell cultures with and without IL-27 exposure.

Cell Population	Cell Culture	Mean	SD	SEM	Median	Min.	Max.	Test Statistic	*p*-Value
Gal-9^+^ total	IL-27–	93.91	4.83	1.17	95.58	81.16	99.42	2.817 ^W^	0.005
	IL-27+	96.55	2.77	0.67	97.06	87.54	99.13		
CD4^+^/Gal-9^+^	IL-27–	14.00	9.05	2.19	12.01	2.40	30.54	1.065 ^W^	0.287
	IL-27+	12.81	7.74	1.88	13.08	1.89	30.27		
CD8^+^/Gal-9^+^	IL-27–	12.65	6.12	1.48	13.89	2.58	21.41	0.355 ^W^	0.723
	IL-27+	12.67	6.63	1.61	13.22	2.53	22.86		
CD19^+^/Gal-9^+^	IL-27–	70.40	15.57	3.78	70.17	46.92	92.02	0.970 ^W^	0.332
	IL-27+	71.80	15.11	3.66	70.42	45.33	94.26		

SD—Standard Deviation, SEM—Standard Error of Mean, ^W^—Wilcoxon test.

## Data Availability

The datasets presented in this article are not readily available because the data are part of an ongoing study. Requests to access the datasets should be directed to Tomasz Wandtke.

## Abbreviations

The following abbreviations are used in this manuscript:

AML	Acute Myeloid Leukemia
APC	Allophycocyanin
CLL	Chronic Lymphocytic Leukemia
FITC	Fluorescein Isothiocyanate
Gal-9	Galectin-9
IFN	Interferon
IL	Interleukin
NFIL3	Nuclear Factor, Interleukin 3 regulated
NK	Natural Killer cells
PBMC	Peripheral Blood Mononuclear Cells
PBS	Phosphate-Buffered Saline
PD-L1	Programmed Death-Ligand 1
PE	Phycoerythrin
PerCP	Peridinin-Chlorophyll-Protein
RT	Room Temperature
SD	Standard Deviation
SEM	Standard Error of Mean
SSC	Side Scatter
T-bet	T-box transcription factor
Tc	T cytotoxic lymphocyte
Th	T helper lymphocyte
TIM-3	T cell Immunoglobulin and Mucin domain-3
Treg	T regulatory lymphocyte
